# Isolation and characterization of exopolysaccharide‐producing strains of *Lactobacillus bulgaricus *from curd

**DOI:** 10.1002/fsn3.905

**Published:** 2019-03-03

**Authors:** Khubaib Ali, Muhammad Huzaifa Mehmood, Muhammad Ahmad Iqbal, Tariq Masud, Mudassir Qazalbash, Shahzad Saleem, Sheraz Ahmed, Muhammad Rizwan Tariq, Waseem Safdar, Muhammad Adnan Nasir, Muhammad Tariq Saeed, Abid Muhammad, Muhammad Naveed Sheas

**Affiliations:** ^1^ Department of Diet & Nutritional Sciences University of Lahore Islamabad Pakistan; ^2^ Faisalabad Medical University Faisalabad Pakistan; ^3^ King Edward Medical University Lahore Pakistan; ^4^ PMAS Arid Agriculture University Rawalpindi Pakistan; ^5^ Department of Biosciences COMSATS University Islamabad Sahiwal Pakistan; ^6^ Department of Food and Nutrition Cholistan University of Veterinary and Animal Sciences Bahawalpur Pakistan; ^7^ Department of Diet & Nutritional Sciences University of Lahore Gujrat Pakistan

**Keywords:** characterization, curd, EPS, isolation, *Lactobacillus bulgaricus*

## Abstract

Curd is the most widespread traditional fermented milk product used by a large population and is a good source of vitamin B, protein, and calcium. In this study, the isolation of exopolysaccharide (EPS)‐producing strains of *Lactobacillus delbrueckii* subsp. *bulgaricus *from curd samples was carried out. Identification of EPS‐producing strains was done by Gram staining, catalase activity, sugar fermentation test, API 50 CHL, and PCR analysis. These EPS‐producing strains were subjected for the estimation of technological properties such as titratable acidity, curdling time, acidification rate, and texture. The strains best in their technological properties were selected for the production of yogurt in combination with EPS‐ or non‐EPS‐producing strains of *Streptococcus thermophilus.* The EPS concentration range was from 41 to 268 mg/L in the yogurt. The highest value of EPS concentration was detected in *S. thermophilus* and non‐EPS‐producing *Lb. bulgaricus* after 14 days of storage.

## 1 INTRODUCTION

Curd analogue to yogurt is an acidified product and considered as more popular among all age‐groups in Indo‐Pak subcontinent. Yogurt was formed by the action of starter cultures containing *Lactobacillus delbrueckii *subsp. *bulgaricus* and *Streptococcus thermophilus *that ferment the milk. During the fermentation of milk, the yogurt starter culture showed symbiotic effect that results in the formation of typical yogurt texture, aroma, and other physiological properties (Bhuiyan, Wadud, Nahar, & Al‐Amin, 2010). Among consumers, yogurt having good texture, high viscosity, and no syneresis was considered as good quality yogurt. Different additives such as gelatin, pectin, and starch were used to obtain yogurt with good rheological properties and without syneresis. Recently, the consumers are reluctant to use additive‐free products. In order to overcome this problem, different natural or biostabilizers, texturizers, and emulsifying agents were used that improve the quality of fermented dairy product (Sharma et al., 2012). Yogurt starter culture is able to produce exopolysaccharide (EPS) during fermentation that plays crucial role in the rheological and physiochemical properties and acts as biostabilizer (Widyastuti & Febrisiantosa, 2014). EPSs are eco‐friendly polymers that are secreted by different strains of bacteria in their surrounding medium and are used in different product formulations. EPSs are of two types: homo‐EPSs, consisting of single monomer, and hetero‐EPSs, consisting of repeating units of different monomers (Vuyst & Degeest, 1999). The use of EPS‐producing starter culture in fermented dairy product acts as an alternative of different stabilizers, and nowadays, people preferred food having natural additives or free of artificial additives (Lin & Chien, 2007).

Exopolysaccharides act as biostabilizer that increase the viscosity with low syneresis and also improve the sensory attributes of fermented dairy product by binding the free water in the product. In this study, the focus of our research was on the production of EPS from *Lactobacillus bulgaricus* (Welman, Maddox, & Archer, 2003). The aim of our study was to select the strain that is able to produce higher EPSs and their utilization in yogurt preparation is able to overcome the problem of syneresis, thereby improving its texture. The use of high amount of EPS‐producing strains will reduce the use of artificial additives and can be comparable with imported starter culture.

## 2 MATERIALS AND METHODS

### 2.1 Materials

The total number of 40 curd samples was collected randomly from local market of Rawalpindi. These samples were collected in sterilized bottles and immediately transferred to the laboratory for further analysis.

### 2.2 Identification of *Lactobacillus bulgaricus*

For the isolation of *L. bulgaricus*, selective medium De Man, Rogosa, and Sharpe (MRS) composed of 1.0% peptone, 1.0% beef extract, and 0.4% yeast extract was used. The colonies produced on MRS agar were examined for morphology, and selected colonies were subjected to biochemical test (API 50 CHL Kit; bioMerieux). Further confirmation of *L. bulgaricus *was done by molecular method with some modification in polymerase chain reaction (PCR) steps using specific primers bulgfor forward primer TCAAAGATTCCTTCGGGATG and bulgrev reverse primer TACGCATCATTGCCTTGGTA (Tabasco, Paarup, Janer, Peláez, & Requena, 2007). Each of the 35 amplification cycles consisted of primer annealing at 60°C for 20 s, primer extension at 72°C for 20 s, and heat denaturation at 94°C for 35 s.

### 2.3 Exopolysaccharide‐producing strain selection

Eight strains of *Lb. delbrueckii *ssp.* bulgaricus* were screened after biochemical and molecular tests from indigenous curd. These isolated strains were subjected for their EPS production by inoculating them into sterilized (121°C for 15 min) 10% skim milk and incubated at 42°C for 24 hr. Then, 2% of fermented sample was added in freshly prepared MRS broth and incubated at 42°C for 24 hr for EPS estimation.

### 2.4 Isolation and quantification of EPS

#### 2.4.1 Isolation

Isolation of EPS was done by the method described by Rimada and Abraham (2003). Ten milliliters of sample was taken from the fermented bottle and heated in boiling water bath at 100°C for 15 min to dissolve the polysaccharides that are attached to cells and to inactivate the enzymes. After cooling, the samples were centrifuged at 15941 g at 20°C for 10 min to remove cells and 17 ml of 85% trichloroacetic acid was added for 100 ml of sample and cooled at 4°C, and then centrifuged at 8,000 rpm for 10 min to remove protein contents from samples. Precipitation of EPS from samples was provided using cold ethanol (−20°C, 1:3). Samples were stored at 4°C for 48 hr and late centrifuged (40°C, 8,000 rpm, for 10 min). Then, the resultant precipitation was dissolved in dH2O and the EPS was defined.

#### 2.4.2 Quantification

Five per cent phenol solution was prepared in water by dissolving 5 g of fresh phenol in dH2O and the volume was made to 100 ml. For the calibration purpose, 1 mg/ml glucose solution and six different standards of glucose were prepared. Four hundred microliters of sample and 400 µl of 5% phenol solution in water were mixed in the glass test tube. A control sample was also prepared in which 400 µl of dH2O and 400 µl of 5% phenol solution in water were used. Then, 2 ml of concentrated sulfuric acid was added to the solution into the tube and left for 10 min. Then, the mixture was shaken and again left for 10 min at 30°C. Then, the samples were run at 490 nm in quartz on 752 UV‐Vis spectrophotometer cuvettes, and the reading was recorded and compared with control. The amount of EPS (mg) was calculated using glucose calibration line (Feldmane, Semjonovs, & Ciprovica, 2013).

### 2.5 Technological screening

The milk (12% reconstituted skim milk) was fermented with identified EPS‐producing *Lb. delbrueckii *ssp. *bulgaricus *strains at 37°C till curd is settled down. Technological parameters such as curdling time, titratable acidity, flavor, and texture and body were evaluated. Titration method was used to evaluate titratable acidity from fermented milk products, and sensory method was used to estimate the curdling time, body and texture, and flavor.

### 2.6 Preparation of yogurt by selected EPS‐producing strains of *Lb. delbrueckii *ssp. *bulgaricus* by using cow milk

Fresh cow milk was obtained from National Agriculture Research Center (NARC), Islamabad. The raw milk contains total solids 12.5%, total protein 3.30%, lactose 4.6%, fat 3.8%, pH 6.65, and titratable acidity 0.14. The raw milk was heated up to 40°C, and their solid contents were increased up to 15% by adding skim milk powder. Then, milk was heated at 90°C for 30 min and cooled to 42°C, and then, milk was divided into four equal portions 200 ml for each and incubated with EPS‐producing strains that were identified in our research and incubated with four different combinations that were as follows:

Treatment A: cow milk inoculated with 1% (*v*/*v*) non‐EPS‐producing *Lb. delbrueckii *ssp.* bulgaricus* plus 1% (*v*/*v*) non‐EPS‐producing *S. thermophilus* as control.
Treatment B: cow milk inoculated with 1% (*v*/*v*) EPS‐producing *Lb. delbrueckii *ssp.* bulgaricus* plus 1% (*v*/*v*) non‐EPS‐producing *S. thermophilus*.
Treatment C: cow milk inoculated with 1% (*v*/*v*) non‐EPS‐producing *Lb. delbrueckii *ssp.* bulgaricus* plus 1% (*v*/*v*) EPS‐producing *S. thermophilus*.
Treatment D: cow milk inoculated with 1% (*v*/*v*) EPS‐producing *Lb. delbrueckii *ssp.* bulgaricus* plus 1% (*v*/*v*) EPS‐producing *S. thermophilus*.

Each portion of cow milk was inoculated with 2% (*v*/*v*) starter cultures; then, milk was transferred into 200 g plastic cups and incubated at 42°C for 4–5 hr until coagulation, and then, cups were cooled down at 4°C and stored for 21 days. Then, the yogurt was analyzed experimentally on weekly basis as chemical or organoleptical. The experiment was repeated three times in duplicate.

#### 2.6.1 Chemical analysis

The pH of yogurt sample was measured by pH meter and combined glass electrode. EPS contents from yogurt sample were determined by the method described by Dubois, Gilles, Hamilton, Rebers, and Smith (1956). One volume of fermented milk was mixed with one volume of 20% (wt/vol) trichloroacetic acid (TCA), and then heated at 100°C for 5 min and centrifuged at 3,500 *g* at 20°C for 10 min. The supernatant was removed after centrifugation and 0.5 volumes of 10% TCA was added and again centrifuged. Aqueous phases were pooled and dialyzed at 4°C against deionized water for 4 days. Then, EPS concentration in the suspension was quantified by phenol–sulfuric acid method and was expressed as glucose equivalent with glucose as a standard.

### 2.7 Rheological measurements

#### 2.7.1 Apparent viscosity

Apparent viscosities of yogurt were measured according to method described by Shihata and Shah (2002). The apparent viscosity was measured on cup at 20°C with a Brookfield viscometer after 1, 7, 14, and 21 days of storage. The spindle used (LV‐SC4‐34 spindle at 4 rpm) in 150 g of yogurt was allowed to rotate for 1 min at 20°C.

#### 2.7.2 Syneresis

Susceptibility of yogurt to syneresis was determined by centrifuging 20 g of sample at 500 rpm for 5 min, weighing the supernatant, and then measuring the amount of supernatant recovered. Percent syneresis was calculated as:

$$\text{\textbackslash{}\% Syneresis =}\;\frac{\text{Volume}\;\text{of}\;\text{supernatant}}{\text{Weight}\;\text{of}\;\text{sample}}\times 100.$$

#### 2.7.3 Sensory evaluation

Twenty trained panelists (14 women and six men, aged 22–45) were asked to evaluate the sensory attributes of yogurt. The ratings were presented on a 9‐point hedonic scale ranging from 9 (“like extremely”) to 1 (“dislike extremely”). Yogurt sensory parameters were evaluated by thickness, smoothness, fermented odor, finished flavor, and taste quality. The yogurts were served to panelists after the cooling process. Result was given on averages of the three trials for each type of yogurt (Sahan, Yasar, & Hayaloglu, 2008).

### 2.8 Statistical analysis

Completely randomized design was used for statistical analysis for experimental data. Statistical analyses were performed using SPSS 14.0 software (SPSS Inc.; Chicago, IL, USA). ANOVA followed by Tukey’s test was used for statistical differences with a level of significance  = 0.05 (Han et al., 2016).

## 3 RESULT AND DISCUSSION

### 3.1 Isolation of EPS‐producing *Lb. delbrueckii *ssp.* bulgaricus strains*

Exopolysaccharide‐producing bacterial culture produced mucoid colonies on milk agar and MRS agar plates. Morphological examination of these colonies was done by Gram staining, and based on their morphology, colonies containing long rods or rods in chains were picked and repetitive streaking method was followed to purify these colonies using the same method for isolation of lactic acid bacteria described by Behare et al. (2010).

#### 3.1.1 Identification of *Lb. delbrueckii *ssp.* bulgaricus*

Biochemical test reveals that 15 isolates were *Lb. delbrueckii *ssp.* bulgaricus *out of 40 isolates collected from indigenous curd and other isolates were *Pediococcus lolli*, *Lactobacillus fermentum*, *Lactobacillus acidophilus*, *Lactobacillus casei*, and* Lactobacillus lactic. *These selected *Lb. delbrueckii *ssp.* bulgaricus *were further identified at molecular level by using specific primer. PCR confirmed eight out of 15 isolates as *Lb. delbrueckii *ssp.* bulgaricus* with amplification product of 252 bp. Similar kind of study was conducted by Tabasco et al. (2007): They used species‐specific primer for suitable identification of *S. thermophilus*, *L. delbrueckii *ssp.* bulgaricus*, *L. acidophilus*, *Lactobacillus paracasei*, and *L. lactic* in culture‐independent analysis of the fermented sample. In the past, Suhartatik, Cahyanto, Rahardjo, Miyashita, and Rahayu (2014) reported the identification of different strains by PCR amplification using similar method.

#### 3.1.2 Isolation and quantification of EPS

Different strains of *Lb. delbrueckii *ssp.* bulgaricus* in MRS medium were able to produce EPS that varies roughly from 120.79 to 175.50 mg/L of yogurt samples. The summarized results shown in Table 1 reveals that L1 starter produced greater quantity of EPS (175.50 mg/L) while L6 produced lower amounts of EPS (120.79 mg/L) in MRS medium. After the previous analysis, the ropy polysaccharide producing strains have ability to produce higher amount of polysaccharide Furthermore, it was also confirmed that there is no correlation between acid productions and curdling time with EPS amount. In the past research, recognition of EPS‐producing strains was carried out by different identification methods: Their EPS concentration was measured by ethanol precipitation followed by phenol–sulfuric acid method, their amount was detected as glucose equivalent by spectrophotometer at 490 nm, and different standards of glucose were used (Han et al., 2016). Similar method was used by Petry, Furlan, Crepeau, Cerning, and Desmazeaud (2000) for the isolation and quantification of EPS produced by *Lb. delbrueckii *ssp.* bulgaricus* in CDM.

**Table 1:** Estimation of acidification rate of *Lactobacillus bulgaricus* strains

S. no.	*Lactobacillus delbrueckii *ssp.* bulgaricus*	EPS in MRS medium (mg/L)
1	L1	175.50 ± 22
2	L2	163.29 ± 6
3	L3	167.90 ± 25
4	L4	152.60 ± 31
5	L5	170.50 ± 5
6	L6	120.79 ± 14
7	L7	143.47 ± 17
8	L8	125.90 ± 3
9	L9	145.60 ± 15
10	L10	135.53 ± 7

Note

EPS: exopolysaccharide; MRS: De Man, Rogosa, and Sharpe.

### 3.2 Technological screening

For the evaluation of technological properties, all the selected EPS‐producing *Lb. delbrueckii *ssp.* bulgaricus *strains were further tested for their curdling time, acidity, and body and texture. Technological properties of selected strains are summarized in Table 2. It was observed that the four strains L1, L3, L5, and L7 showed better results in flavor, and body and texture of final product as compared to other tested strains. There was inverse relationship between curdling time and titratable acidity but no clear‐cut relationship with EPS production because EPS‐producing genes were located on chromosomes, so EPS production varies from strain to strain. The technological parameters that were greatly affected by EPS production were body and texture, and production of EPS improved these parameters.

**Table 2:** Comparison of technological screening of exopolysaccharide (EPS)‐producing strains of *Lactobacillus delbrueckii *ssp. *bulgaricu*s

EPS‐producing *Lb. delbrueckii *ssp. *bulgaricu*s strains	Types of EPS		Acidity (% lactic acid)	Curdling time (hr)	Flavor, body, and texture
	CPS	RPS			
L1	+	+++	0.71	6	Good body and texture, pleasant flavor
L2	+	+	0.68	8	Good body and texture but acidic
L3	+	+++	0.70	7	Good body and texture, pleasant flavor
L4	+	−	0.66	8	Poor body and texture, pleasant flavor
L5	+	+++	0.68	5	Good body and texture, pleasant flavor
L6	+	−	0.62	9	Poor body and texture, pleasant flavor
L7	+	++	0.69	5	Good body and texture, pleasant flavor
L8	+	−	0.65	7	Good body and texture, pleasant flavor
L9	+	++	0.69	6	Good body and texture, pleasant flavor
L10	+	−	0.84	8	Good body and texture, pleasant acidic

### 3.3 Preparation of yogurt by selected EPS‐producing strains of *Lb. delbrueckii *ssp. *bulgaricus* by using cow milk

The chemical composition of cow’s fermented product made with EPS‐ and non‐EPS‐producing starter culture during storage of 21 days at 4°C is presented in Table 3. There is a significant difference (*p* < 0.05) among TA% of all treatments. In all treatment, TA% gradually increased with increasing storage periods up to 21 days. It was observed that the concentration of TA% in treatment A was maximum after 21 days of storage and subsequently decreased on 14, 7, and 1 day of storage. The reason behind that was the yogurt made with non‐EPS‐producing starter culture produces more TA% because non‐EPS‐producing starter cultures utilize carbohydrate as their energy source as well as their carbon source for lactic acid production. The EPS concentration ranges from 41 to 268 mg/L in the yogurt that was made by EPS‐ and non‐EPS‐producing mixed starter culture. The highest value of EPS concentration was detected in treatment C that was made with EPS‐producing *S. thermophilus* (isolated from different curd samples) and non‐EPS‐producing *Lb. bulgaricus* after 14 days of storage, and there was a significant difference (*p* < 0.05) between EPS, then reduced after more storage time. Yogurt produced by treatment C produced maximum EPS because the ability of polymer forming thermophilic streptococci is higher than that of the *lactobacilli.* Similar kind of study was conducted by Feldmane et al. (2013): They used different commercial yogurt starter cultures with tested EPS‐producing starter cultures for the fermentation of milk, and then, EPS content was estimated that varies roughly from 144.02 to 440.09 mg/L in yogurt sample. In past studies, Gürsoy, Durlu‐Özkaya, Yildiz, and Aslim (2010) also estimated that maximum EPS concentration was recorded on the 11th day of storage that was inoculated with commercial starter culture mixed with *Lb. delbrueckii *ssp.* bulgaricus.*

**Table 3:** Determination of chemical and rheological properties of yogurt prepared with non‐ exopolysaccharide (EPS)‐producing (control) and EPS‐producing cultures during storage period at 4 ± 1°C

Treatment	Storage (days)	Titratable acidity	EPS g/kg	Viscosity (cp)	Firmness (g)	Syneresis (ml/100 g)
Lb(NEPS) + Str (NEPS) (A)	1	0.75^j^ ± 0.01	45.3^n^ ± 0.58	566^l^ ± 4.90	68.0^a^ ± 0.1	42.2^a^ ± 0.56
	7	0.79^hij^ ± 0.01	53.7^m^ ± 2.31	586^k^ ± 5.75	67.6^a^ ± 0.5	39.0^b^ ± 0.39
	14	0.93^cd^ ± 0.06	61.3^l^ ± 1.13	640^j^ ± 10.87	62.2^de^ ± 0.6	38.0^b^ ± 0.56
	21	1.02^a^ ± 0.05	41.3^o^ ± 0.57	630^j^ ± 6.70	58.2^f^ ± 0.6	37.0^c^ ± 0.99
Lb(EPS) + Str(NEPS) (B)	1	0.78^ij^ ± 0.03	161.9^k^ ± 1.00	839^J^ ± 3.99	66.6^b^ ± 0.5	32.1^e^ ± 0.56
	7	0.83^ghi^ ± 0.01	185.7^g^ ± 0.57	880^i^ ± 5.70	65.6^c^ ± 0.5	30.2^f^ ± 0.58
	14	0.86^efg^ ± 0.02	198.0^e^ ± 1.73	935^g^ ± 7.60	63.0^d^ ± 0.2	27.3^h^ ± 0.58
	21	0.93^bc^ ± 0.04	167.0^j^ ± 0.01	910^h^ ± 4.55	62.1^e^ ± 0.1	29.3^ef^ ± 0.58
Lb(NEPS) + Str(EPS) (C)	1	0.77^ij^ ± 0.03	214.0^c^ ± 4.00	1,255^e^ ± 3.55	50.2^h^ ± 0.5	24.2^j^ ± 0.56
	7	0.83^efgh^ ± 0.03	252.3^b^ ± 2.88	1,315^d^ ± 5.70	51.2^g^ ± 0.5	21.6^k^ ± 0.56
	14	0.87^de^ ± 0.05	268.2^a^ ± 1.14	1,365^b^ ± 5.70	48.5^i^ ± 0.5	20.3^l^ ± 0.57
	21	0.98^b^ ± 0.03	191.9^i^ ± 2.50	1,360^b^ ± 5.70	43.6^j^ ± 0.5	25.9^i^ ± 1.00
Lb(EPS) + Str(EPS) (D)	1	0.78^ij^ ± 0.03	181.0^h^ ± 1.52	1,150^t^ ± 2.80	39.3^i^ ± 0.3	28.0^gh^ ± 0.98
	7	0.83^fgh^ ± 0.02	208.4^d^ ± 2.84	1,265^e^ ± 9.50	41.1^k^ ± 0.2	27.6^gh^ ± 0.54
	14	0.87^def^ ± 0.04	190.4^f^ ± 0.58	1,330^c^ ± 5.75	35.7^lm^ ± 0.6	25.3^ij^ ± 0.52
	21	0.98^b^ ± 0.04	175.3^i^ ± 1.56	1,385^a^ ± 3.95	36.5^m^ ± 0.7	28.6^fg^ ± 0.58

Note

^abcde^Mean followed by different letters in the same column is significantly different (*p* < 0.05).

The rheological parameters such as change in viscosity, firmness, and syneresis of cow milk yogurt made with EPS‐ or non‐EPS‐producing starter culture were determined after 21 days of storage as summarized in Table 3. The highest value of viscosity was found in treatment D during maximum storage of 21 days, and there was a significant difference (*p* < 0.05) between viscosities of yogurt after 21 days of storage and after 1, 7, and 14 days of storage. This is due to the ability of EPS interaction with milk proteins. The greater the amount of EPS, the greater will be the interaction with proteins present in the milk, so the viscosity of yogurt increased with EPS‐producing starter culture. It was also affected by the amount of EPS and their molecular characteristics. Similar kind of study was reported by Han et al. (2016) and proved that yogurt made with EPS‐producing strains showed more viscosity as compared to yogurt made with non‐EPS‐producing strains and little increase in EPS can affect more viscosity and adhesiveness. The viscosity of yogurt sample increased due to the presence of EPS‐producing strains in starter culture as reported by Gürsoy et al. (2010).

The firmness of yogurt made with non‐EPS‐producing starter culture was higher in control sample as compared to yogurt made with ropy EPS‐producing starter cultures. The highest value of firmness was found after 1 day of storage of fresh control sample treatment, and there was a significant difference (*p* < 0.05) between readings of different days of storage. Similar results were reported by Gürsoy et al. (2010) that the gel firmness was not affected by EPS‐producing starter culture. Results also showed that yogurt made with EPS‐producing starter culture showed lower syneresis as compared to yogurt made with non‐EPS‐producing starter culture (control). This result showed that syneresis of fermented milk product with EPS‐producing starter culture depends upon the ability of EPS to bind water and that ability is affected by its type and concentration in the product and their interaction and distribution with protein network. Furthermore, longer fermentation time of cow milk allowed more structural rearrangement due to this weak structure formed and spontaneous syneresis increase. The collected data showed that sensory quality acceptance and the visual appearance of yogurt made with EPS‐producing strains were same as those of yogurt produced by non‐EPS‐producing strains and the gels were smooth and free of syneresis. The yogurt made with non‐EPS‐producing starter culture (control) had significantly (*p* < 0.05) lower rating for texture and body, color, flavor, and appearance and overall score, due to whey off on the fermented milk surface (Table 4). Moreover, rich mouthfeel and good acid taste were observed in treatments B, C, and D, and flavor was also good after 14 days of storage period, but the appearance and acceptability decreased due to whey off on the fermented milk surface.

**Table 4:** Sensory evaluation of yogurt prepared with non‐exopolysaccharides (EPS)‐producing (control) and EPS‐producing cultures during storage period at 4 ± 1°C

Treatment	Storage (days)	Flavor (max 10 points)	Body and texture (max 5 points)	Appearance and color (max 5 points)	Overall acceptability score (20)
Lb(NEPS) + Str (NEPS) (A)	1	7.7^b^ ± 0.75	3.1^e^ ± 0.89	3.4^b^ ± 0.52	12.3^c^ ± 1.25
	7	7.6^b^ ± 0.52	3.1^e^ ± 0.69	3.7^b^ ± 0.47	14.4^c^ ± 0.53
	14	8.1^ab^ ± 0.38	3.4^de^ ± 0.53	3.4^b^ ± 0.52	15.0^c^ ± 0.82
	21	7.7^b^ ± 0.75	3.3^de^ ± 0.48	3.5^b^ ± 0.51	14.5^c^ ± 1.49
Lb(EPS) + Str(NEPS) (B)	1	7.9^b^ ± 0.68	4.3^abc^ ± 0.49	4.4^a^ ± 0.53	16.3^b^ ± 1.24
	7	8.3^ab^ ± 0.78	4.4^ab^ ± 0.52	4.5^a^ ± 0.53	17.2^ab^ ± 1.10
	14	8.4^ab^ ± 0.78	3.9^bcd^ ± 0.69	4.4^a^ ± 0.53	16.5^b^ ± 1.11
	21	8.2^ab^ ± 0.64	3.7^cde^ ± 0.48	4.4^a^ ± 0.53	16.4^b^ ± 1.51
Lb(NEPS) + Str(EPS) (C)	1	8.1^ab^ ± 0.90	4.7^a^ ± 0.49	4.5^a^ ± 0.53	17.4^ab^ ± 1.12
	7	8.9^a^ ± 0.37	4.6^a^ ± 0.52	4.9^a^ ± 0.39	18.3^a^ ± 0.95
	14	8.3^ab^ ± 0.75	4.6^a^ ± 0.52	4.6^a^ ± 0.53	17.4^ab^ ± 0.97
	21	8.1^ab^ ± 0.68	4.9^a^ ± 0.37	4.6^a^ ± 0.53	17.6^ab^ ± 0.52
Lb(EPS) + Str(EPS) (D)	1	8.0^ab^ ± 0.57	4.3^abc^ ± 0.48	4.6^a^ ± 0.53	16.8^b^ ± 0.98
	7	8.1 ± 0.89	4.7^a^ ± 0.49	4.6^a^ ± 0.38	17.5^ab^ ± 1.57
	14	8.3^ab^ ± 0.52	4.6^a^ ± 0.52	4.6^a^ ± 0.53	17.6^ab^ ± 0.78
	21	8.0^ab^ ± 0.79	4.7^a^ ± 0.48	4.6^a^ ± 0.53	17.2^ab^ ± 1.11

Note

^abcde^Mean followed by different letters in the same column is significantly different (*p* < 0.05).

The overall acceptability scores of the yogurt made by EPS‐producing starter culture were higher and acceptable in treatment C as compared to control treatment. The flavor of yogurt was more preferred by panel of consumer that was made with non‐EPS‐producing starter culture as the score was higher (*p* < 0.05). This difference is due to the presence of more acetaldehyde contents in yogurt that was made with non‐EPS‐producing starter culture.

## 4 CONCLUSION

Yogurt is a fermented milk product and gains more popularity in every age of people due to their unique flavor. Yogurt contains a heterogeneous mixture of starter culture that ferments the milk and produces different bioactive compounds and biostabilizers. In this research work, 15 EPS‐producing *Lb. delbrueckii *ssp. *bulgaricus *were isolated. Different PCR conditions were fixed to get the proper results. EPS‐producing bacterial strains act as biostabilizer and have the ability to enhance the rheological properties of yogurt, and it is an innovative idea to replace artificial stabilizers such as gums, gelatin, pectin with these natural EPS‐producing bacterial strains.

## CONFLICT OF INTEREST

The authors declare that they do not have any conflict of interest.

## ETHICAL APPROVAL

This study does not involve any human or animal testing.
